# Level of physical activity among nurses and its associated factors: A cross-sectional study

**DOI:** 10.1177/10519815251386437

**Published:** 2025-10-17

**Authors:** Hassan Alrabbaie, Khader Almhdawi, Roger Goldstein, Marla Beauchamp, Dina Brooks

**Affiliations:** 1 School of Rehabilitation Sciences, Faculty of Health Sciences, McMaster University, Hamilton, Ontario, Canada; 2 Department of Rehabilitation Sciences, Faculty of Applied Medical Sciences, Jordan University of Science and Technology, Irbid, Jordan; 3 Department of Respiratory Medicine, West Park Healthcare Centre, Toronto, Ontario, Canada; 4 Department of Physical Therapy, Faculty of Medicine, University of Toronto, Toronto, Ontario, Canada; 5 Rehabilitation Sciences Institute, School of Graduate Studies, University of Toronto, Toronto, Ontario, Canada; 6 Department of Medicine, Faculty of Medicine, University of Toronto, Toronto, Ontario, Canada

**Keywords:** occupational health, health promotion, night shift, sleep, musculoskeletal pain, mental health

## Abstract

**Background:**

Physical activity is essential for preventing chronic disease and maintaining overall health. However, hospital nurses may face challenges maintaining adequate physical activity due to demanding work schedules and occupational stressors.

**Objective:**

To examine the levels of physical activity among hospital nurses in Jordan and to identify demographic, occupational, and health-related factors associated with physical activity.

**Method:**

A cross-sectional survey was conducted among 750 nurses across Jordanian hospitals, with 597 respondents (80% response rate). Validated self-administered questionnaires were used to assess demographic, work characteristics, psychological well-being, sleep quality, musculoskeletal pain, and physical activity. Descriptive statistics summarized participant characteristics, and multiple linear regression was performed to identify independent associations with physical activity levels.

**Results:**

The mean age of participants was 32.1 years, and average work hours were 43.4 h per week. Approximately 31% of nurses report moderate physical activity levels, while 40% reported high physical activity levels. Higher physical activity levels were independently associated with longer work hours (β = 46.1; 95% CI: 1.9 to 90.2), more frequent night shifts (β = 163.8; 95% CI: 11.8 to 315.7), and more musculoskeletal pain sites (β = 254.9; 95% CI: 171.3 to 338.7). Having a chronic disease was significantly associated with lower physical activity (β = -1384.1; 95% CI: −2443.5 to −324.1).

**Conclusion:**

Most nurses met recommended physical activity levels, and their engagement in physical activity was influenced by work demands and health status. Workplace health promotion initiatives should consider these factors to effectively support and sustain physical activity among hospital nurses.

## Introduction

Physical activity refers to any bodily movement generated by skeletal muscles that requires the expenditure of energy.^1^ Regular physical activity has consistently been demonstrated to lower the likelihood of developing chronic conditions, extend lifespan, promote well-being, and enhance the health-related quality of life.^2–5^ Moreover, higher physical activity has been associated with enhanced work productivity and a decrease in absence from the workplace.^6–8^ The World Health Organization (WHO) advises adults to achieve a minimum of 150 min of moderate-intensity physical activity or 75 min of vigorous-intensity physical activity per week or an equivalent combination of both activity levels, reaching a total of 600 metabolic equivalent task (MET) minutes per week.^9,10^ One suggested approach to improving physical activity involves leveraging workplace settings to encourage employees to participate in regular physical activity.^7^

Better adherence to personal health practices among healthcare professionals may influence the quality of care they provide to their patients.^11,12^ Fie et al. demonstrated that nurses with positive attitudes towards physical activity and who engage in high levels of activity are more likely to encourage their patients to be active than less active nurses.^13^ Moreover, patients may perceive nurses and doctors as more credible when they adhere better to their health promotion guidance.^13,14^

Working in nursing involves facing a demanding work setting characterized by irregular shifts and rotations.^15,16^ Additionally, this occupation often requires extended working hours of up to 12 h, including daytime and nighttime shifts, and performing physically demanding tasks that can impose negative health impacts on nurses.^15,17–19^ Because of the nature of their profession, nurses face an increased susceptibility to musculoskeletal injuries that might impact productivity and act as a barrier to engaging in physical activity.^20–22^ Previous studies have shown that musculoskeletal symptoms are negatively associated with physical activity levels among nurses.^22,23^ However, limited research has explored the connection between musculoskeletal symptoms and physical activity levels among nurses.

Various factors have been identified as associated with nurses’ physical activity levels.^7^ Among demographic factors, age has received considerable attention in previous studies,^24–26^ but findings have been inconsistent. For instance, Zapka and colleagues observed that nurses aged 40 and above demonstrated greater engagement in physical activity compared to their younger counterparts.^25,27^ Conversely, Almajwal et al. found that nurses aged 50 and above reported lower levels of physical activity.^26^ Additionally, factors like BMI, smoking status, and overall health have been linked to physical activity levels among nurses.^7^ Physical activity levels are negatively associated with higher BMI^28^ and smoking^27,29^ among nurses. Furthermore, there is a positive association between nurses’ self-reported general health status and physical activity levels.^7,28^ Additionally, Han et al. showed that nurses who do not engage in regular exercises (and frequently work night shifts) are more likely to experience sleeping disorders.^30^

Work-related variables, such as shift patterns, occupational roles, working hours, job requirements, and occupational stress, have also been found to be associated with physical activity levels.^7^ Specifically, engaging in the night shift has been consistently linked to lower overall physical activity levels.^17,26,31^ Nam et al. used the Job Content Questionnaire^32^ and found that nurses in positions with low job demands and limited job control exhibit significantly lower levels of physical activity.^17^ On the other hand, job stress has been positively associated with physical activity levels during working hours.^25^

Nurses who participate in regular physical activity tend to have lower incidences of psychological issues such as depression, anxiety, and stress, as well as improved overall mental well-being.^33–35^ Maintaining an active lifestyle can be an effective strategy for nurses to combat psychological distress and enhance their overall well-being.^34,36^

Despite the growing body of international research exploring physical activity among nurses and its determinants, there remains a significant gap regarding comprehensive, context-specific investigations within the Jordanian healthcare system. To date, no studies have systematically examined the interplay between physical activity levels and a broad range of demographic, occupational, psychological, and musculoskeletal factors among nurses in Jordan. Furthermore, the simultaneous assessment of psychological distress (using the Depression, Anxiety, and Stress Scale [DASS]), sleeping quality (using the Pittsburgh Sleep Quality Index [PSQI]), and musculoskeletal symptoms in relation to physical activity is novel in the Jordanian context. By addressing these gaps, our study provides the first holistic analysis of the determinants of physical activity among Jordanian nurses, offering new insights that can inform targeted interventions and health promotion strategies in this population.

Therefore, this study aims to determine the factors influencing physical activity levels among Jordanian nurses. Our primary objectives were:

1. To describe physical activity levels among nurses in Jordan.
2. To determine the association between physical activity and demographic and anthropometric factors, including age, BMI, smoking status, and chronic disease among nurses in Jordan.
3. To examine the association between physical activity levels in Jordanian nurses and psychological conditions such as depression, anxiety, and stress as measured by the Depression, Anxiety, and Stress Scale (DASS).
4. To examine the association between physical activity and sleep among Jordanian nurses as measured by the Pittsburgh Sleep Quality Index (PSQI).

Our secondary objective was to explore the association between physical activity and self-reported occupational characteristics as well as musculoskeletal symptoms.

## Methods

### Design and sample

This study employed a cross-sectional design. Invitations were sent to approximately 750 registered nurses across various hospitals in Jordan to participate in the research. The inclusion criteria were being a nurse who worked full-time for at least 12 months, being between 22 and 60 years old (the retirement age in Jordan), and engaging in shift work at least two shifts per week. We adopted exclusion criteria from previous studies, which include current pregnancy and any health conditions that make physical activity unsuitable.^37,38^

Jordan's main geographical regions (north, middle, and south) are served by governmental, military, and private hospitals, constituting the country's primary types of large tertiary referral hospitals.

### Ethics and procedures

This study was conducted with the approval of the Institutional Review Board (IRB) at Jordan University of Science and Technology, under approval number 50/117/2018. All participants were recruited from July 2018 to January 2019. The research team travelled to each hospital to inform hospital administrators and nursing leaders about the study and to distribute posters throughout nurses’ stations and information boards. The Posters contained brief descriptions of the study and contact information for the research team. The hospital administrators and nursing leaders helped distribute the paper-based questionnaires to nurses. Before data collection, and to ensure compliance with ethical standards, participants reviewed and signed the IRB-approved consent forms acknowledging their agreement to participate. Nurses who agreed to participate were given the option to either fill out the questionnaire on the same working day or hand it over on their next working shift.

## Measures

### Demographic and anthropometric measures:

Demographic and anthropometric information was collected using self-reported responses. This included age (in years), sex (male or female), weight, height (used to calculate BMI), smoking status (including the number of cigarettes smoked), and the presence of chronic diseases.

### Work characteristics measures:

Work characteristics included type of hospital (military, governmental or private), geographical region (north, middle, south), department (intensive care unit, pediatric ward, operating room, surgical ward, and internal ward), years of experience, work hours per week, number of shifts per week, number of night shifts, and number of day shifts. These variables were collected using self-reported responses.

### Physical activity measures:

The primary outcome of physical activity was assessed using the International Physical Activity Questionnaire (IPAQ-SF), which includes seven items. This questionnaire queried participants about the duration of time spent engaging in various activities over the past week, including walking, moderate activity, vigorous activity, and sitting. IPAQ scoring methods included both categorical and continuous scoring. Continuous scores are expressed in terms of MET (Metabolic Equivalent of Task) – min/week, with specific MET values assigned to different activity levels: walking (3.3 MET), moderate intensity (4.0 MET), and vigorous intensity (8.0 MET).^39^ Participants’ total scores were calculated by summing the duration and frequency of all activity levels over the 7 days to derive a total MET–min/week. Categorical scoring categorizes participants into three activity levels: low (below 600 MET-min/week), moderate (600–3000 MET-min/week), and high (at least 3000 MET-min/week).^9,40–42^ Extensive psychometric evaluations have shown the validity and reliability of the IPQA-SF for assessing physical activity and inactivity levels among adults aged 18 to 65 in various settings. Test-retest reliability has indicated good stability (α < .80).^39,43,44^

### Psychological measures:

Psychological factors were assessed using the Depression Anxiety Stress Scale (DASS-21), a short version of the original DASS-42. It is a reliable tool for measuring mental states, including depression, anxiety, and stress, in both clinical and non-clinical adult populations.^45^ The DASS-21 consists of 21 items divided into three self-reported scales, each with 7 items. These items are rated on a Likert scale from 0 to 3 (0: “did not apply to me at all,” 1: “applied to me some degree or some of the time,” 2: “applied to me a considerable degree or a good part of the time,” and 3: “applied to me very much or most of the time”). Scores for depression, anxiety, and stress are calculated by summing the relevant items. Since the DASS-21 is condensed from the 42-item DASS, scores for each subscale are doubled to determine the final score. Established cut-off scores of 9 for depression, 7 for anxiety, and 14 for stress. Higher scores indicate the presence of mild to extremely severe mental health symptoms.^46^ According to the manual, these scores are categorized as: “normal,” “mild,” “moderate,” “severe,” or “extremely severe”.^45,47,48^

### Sleep quality measures:

Sleep quality was assessed using the Pittsburgh Sleep Quality Index (PSQI). This tool assesses both the quality and pattern of sleep over the preceding month.^49^ Cumulative scores yielded a global score indicating sleep quality, with higher scores indicating poorer sleep quality.^49^ A total PSQI score of 5 or above signifies inadequate sleep quality, with a diagnostic sensitivity of 90% and specificity of 67%.^50^

### Musculoskeletal measures:

Musculoskeletal symptoms were self-reported using the Nordic Musculoskeletal Questionnaire (NMQ).^51^ The NMQ is a well-validated tool commonly used for studying work-related musculoskeletal symptoms.^52,53^ The questionnaire includes a diagram of the body highlighting 9 anatomical regions, such as shoulders and hips. Respondents indicated a yes/no response whether they experienced job-related pain or discomfort in the past 12 months. If they answered “yes,” they further indicated whether the symptoms prevented them from working for a day and whether they sought medical attention for this issue. The NMQ results were used to determine the prevalence of work-related symptoms by body region and, if present, how often these symptoms led to work absence or healthcare utilization.^51,54^

## Statistical analysis

Sample size calculations were conducted using G*power 3.1 software, considering approximately 20 potential factors in the model. It was determined that a minimum of 222 participants was required to achieve adequate statistical power of 80% or higher. Descriptive statistics were used to summarize baseline data, with continuous variables (such as age, BMI, sleep quality index (PSQI), depression, anxiety, and stress (DASS), work hours per week, and experience) reported as mean and standard deviation. Categorical variables (such as sex, smoking status, hospital type, and work department) were summarized using frequencies and percentages.

Multiple linear regression analyses were conducted to identify factors associated with physical activity levels. The predictor variables were entered into the analysis in blocks. The first block included anthropometric and demographic variables such as age (years), sex, BMI, number of cigarettes smoked per day, and the presence of at least one chronic disease. The second block focused on work characteristics, including years of experience, weekly work hours, and the number of night shifts. Psychological variables: levels of depression, anxiety, and stress were included in the third block. Sleep quality was also assessed using the global sleep quality score from the Pittsburgh Sleep Quality Index (PSQI), and musculoskeletal symptoms were evaluated based on the number of pain sites reported over the past twelve months. These variables were analyzed to determine their associations with physical activity levels.

The overall fit of the regression models was assessed using the R² statistic, which measures the proportion of variance in physical activity levels explained by the predictor variables. Regression coefficients and a 95% confidence interval (CI) are reported for each predictor. Statistical significance was determined by a threshold p < 0.05. Statistical analyses were conducted using IBM SPSS Statistics version 25.^55^

## Results

### Participant's characteristics

**Figure 1.**
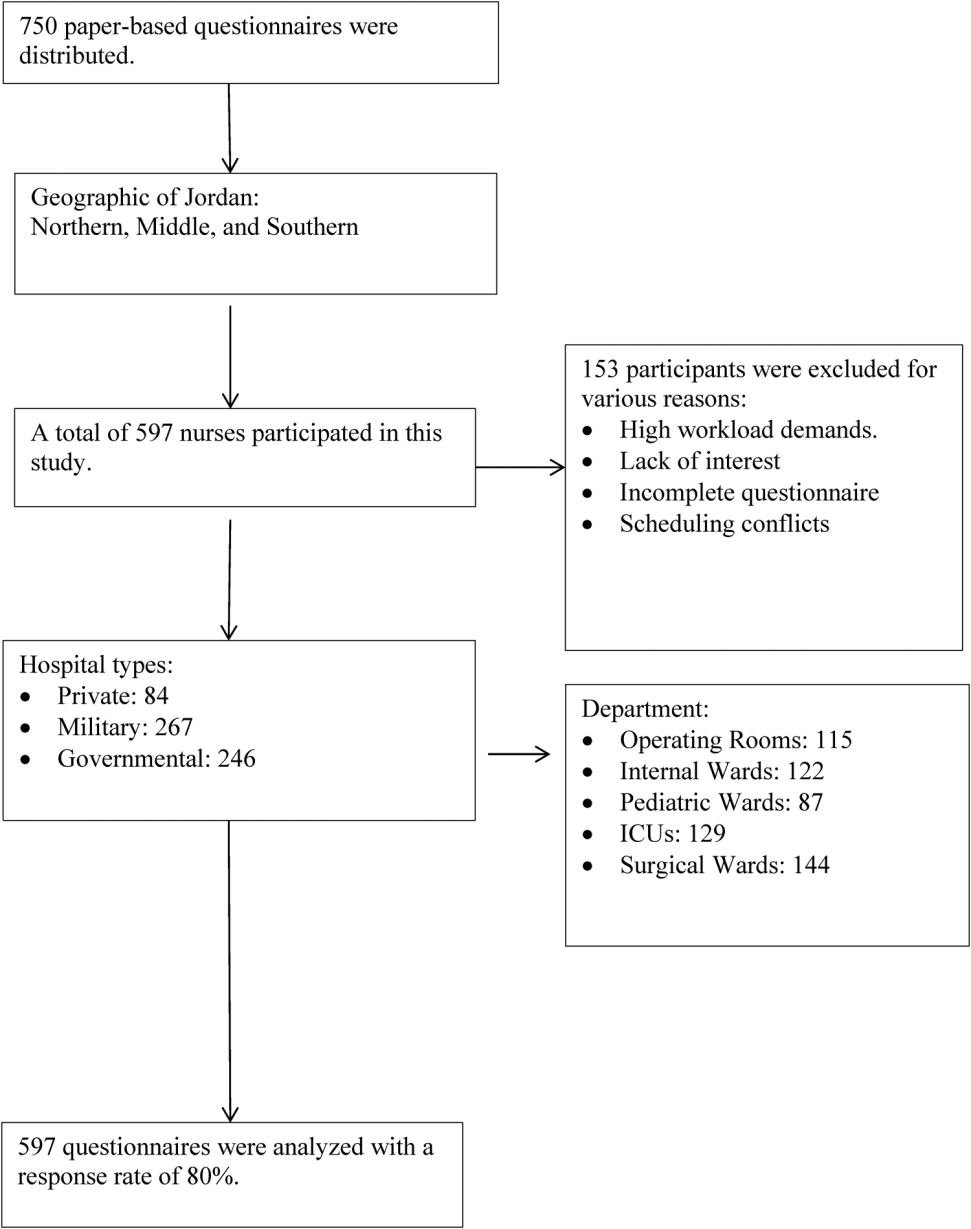
Flow diagram of participant recruitment and response rate across hospitals in Jordan.

We recruited 750 nurses across diverse working sites, ensuring representation from all geographic regions of Jordan, spanning the Northern, Middle, and Southern areas. In total, 597 nurses completed the questionnaire, with a response rate of 80%. Reasons for non-participation included scheduling conflicts, workload demands, or lack of interest (Figure 1).

Participants had a mean age of 32.1 years (± 5.7), with females accounting for 53% of the sample. The average BMI of participants was 25.1 kg/m² (± 3.8), and they had an average of 9.3 years of work experience (± 5.4). Employment was distributed across private 14%, military 45%, and governmental 41% sectors. Geographically, participants were distributed as follows: 49% from the North region, 39% from the Central region, and 12% from the South region.

A quarter of the participants worked in surgical wards. The average number of work hours per week was 43.4 h (± 4.7), with an average of 1.3 night shifts (±1.3). Additionally, 27% of participants were cigarette smokers, with an average consumption of 5.8 cigarettes per day (±10.5) (Table 1).

**Table 1.** Work characteristics and demographics of the study participants (N = 597).

	Mean (SD)	n (%)
Age	32.1 (5.7)	
Experience /Years	9.3 (5.4)	
Sex		Female: 316 (53%)
BMI classification	25.1(3.8)	Underweight: 7 (1.2%) Healthy Weight: 297 (49.7%) Overweight: 233 (39%) Obesity: 60 (10.1%)
Smoking Cigarette number/Day	5.8 (10.5)	162 (27.1%)
Presence of at least one chronic disease		20 (3.4%)
Region		Northern: 293(49.1%) Central: 232 (38.8%) South:72 (12.1%)
Hospital type		Governmental: 246 (41.2%) Private: 84 (14.1%) Military: 267 (44.7%)
Working department		Pediatric ward: 87 (14.6%) Operating room: 115 (19.3%) Internal ward: 122 (20.4) ICU: 129 (21.6%) Surgical ward: 144 (24.1%)
Work hours per week	43.4 (4.7)	
Shifts number per week	5 (.68)	
Day shifts number per week	3.8 (1.5)	
Night shifts number per week	1.3(1.3)	

BMI: Body Mass Index, ICU: Intensive Care Unit, SD: standard deviation, n: number

### Physical activity, sleep quality, and psychological factors levels

Analysis of physical activity levels revealed that 28% of Jordanian nurses had low physical activity, 31% moderate physical activity, and 40% high physical activity according to IPAQ-SF criteria, with an average sum of MET-min/week 2809 (± 2393). Based on the guidelines for physical activity, 71% of participants (those with moderate and high physical activity) met or exceeded the recommended threshold levels of physical activity (600 MET-min/week), while 28% fell below this standard (Table 2). Sixty-eight percent exhibited poor sleep quality, with PSQI scores surpassing five. The mean scores of the Depression Anxiety Stress Scale (DASS-21) indicated a moderate level of severity of depression, anxiety, and stress among participants. Additionally, 91% reported experiencing musculoskeletal symptoms within the past twelve months (Table 3).

**Table 2.** Levels of physical activity (IPAQ-SF) (N = 597).

	Mean (SD)	N (%)
The sum of METs-min/week	2809.6 (2393.3)	597 (100%)
Low PA		170 (28.5%)
Moderate PA		187 (31.4%)
High PA		240 (40.1%)

IPAQ-SF: International Physical Activity Questionnaire, METs: Metabolic Equivalent of Tasks, SD: standard deviation, PA: physical activity

**Table 3.** Psychological well-being (DASS-21), sleep quality (PSQI), and Musculoskeletal symptoms (NMQ).

Measure	Mean (SD)	n (%)
Depression	14.5 (9.4)	
Anxiety	12.9 (8.8)	
Stress	18.7 (9.7)	
PSQI SCORE <5	7.8 (3.8)	407 (68.2%)
12-Month Musculoskeletal symptoms (NMQ)		543 (91%)

(DASS-21): Depression Anxiety Stress Scale, PSQI: Pittsburgh Sleep Quality Index, NMQ: Nordic Musculoskeletal Questionnaire, n: number

### Factors associated with physical activity level

Several assumptions were evaluated to ensure the validity of the multiple regression analysis. The normal probability plot of standardized residuals and the scatterplot of standardized residuals versus standardized predicted values showed that the assumptions of normality, linearity, and homoscedasticity of residuals were satisfied. All Variance Inflation Factor (VIF) values were below 5, indicating no significant multicollinearity. Thus, all the assumptions for linear regression were met.

In the **anthropometric and demographic model**, the presence of at least one chronic disease was associated with a lower level of physical activity (β = -1095.3; 95% CI: −2192.9 to 2.3). However, no associations were found between physical activity levels and age (β = -30.4; 95% CI: −67.3 to 6.5), sex (β = -235.5; 95% CI: −710.1 to 239.1), BMI (β = -27.8; 95% CI: −85.7 to 30.1), or the number of cigarettes smoked per day (β = -10.4; 95% CI: −31.9 to 11.2). The model explained 1.9% of the variance in physical activity levels (R² = 0.019)

In the **work characteristics model**, nurses who worked more hours per week were positively associated with physical activity level (β = 67.8; 95% CI: 23.7 to 111.8). In contrast, neither years of experience (β = -32.8; 95% CI: −71.6 to 6.1) nor the number of night shifts (β = 132.7; 95% CI: −23.1 to 288.6) showed associations with physical activity levels. This model explained 3.1% of the variance in physical activity levels (R² = 0.031)

In the **psychological model**, depression (β = 16.6; 95% CI: −18.1 to 51.2), anxiety (β = 25.8; 95% CI: −9.2 to 60.8), and stress (β = -7.6; 95% CI: −39.5 to 24.4) were not associated with physical activity levels. The model explained 1.6% of the variance of physical activity levels (R² = 0.016)

In the **global sleep quality model**, nurses with higher global sleep quality scores (measured by the PSQI) were associated with increased physical activity levels (β = 77.2; 95% CI: 24.9 to 129.5). The model explained 1.4% of the variance of physical activity level (R² = 0.014)

In the **musculoskeletal symptoms model**, a greater number of pain sites in the past twelve months was positively associated with physical activity levels (β = 256.2; 95% CI: 182.7 to 331.2). The model explained 7.2% of the variance of physical activity levels (R² = 0.072).

In the **full model**, which included variables from all the individual models (anthropometric and demographic, work characteristics, psychological, global sleep quality, and musculoskeletal symptom), the number of work hours per week (β = 46.1; 95% CI: 1.9 to 90.2), the number of night shifts (β = 163.8; 95% CI: 11.8 to 315.7), and the number of pain sites in the past twelve months (β = 254.9; 95% CI: 171.3 to 338.7) were positively associated with physical activity levels. Conversely, the presence of at least one chronic disease was negatively associated with physical activity levels (β = -1384.1; 95% CI: −2443.5 to −324.1). The complete model explained 12.2% of the variance of physical activity levels (R² = 0.122). Detailed results are presented in Table 4.

**Table 4.** Summary of multiple linear regression models for factors associated with physical activity using the sum of MET-min/week as a dependent variable.

Partial models					Full model, R²= 0.122		
Block	Variable	β	95% CI	p-value	β	95% CIs	P-value
Anthropometric and demographic R²= 0.019	Age	−30.4	−67.3–6.5	0.106	−2.3	−83.7–79.1	0.956
	Sex	−235.5	−710.1–239.1	0.330	−412.5	−877.4–52.4	0.082
	BMI	−27.8	− 85.7–30.1	0.346	−46.5	−102.2–9.1	0.101
	Cigarette number/day	−10.4	−31.9–11.2	0.345	−5.8	−26.7–15.1	0.586
	Presence of at least one chronic disease	−1095.3	−2192.9–2.3	0.050*	−1384.1	−2443.5 −324.1	0.011*
Work characteristics measures. R² = 0.031	Experience/year	−32.8	−71.6–6.1	0.097	−16.5	−106.2–73.2	0.718
	Work hours per week	67.8	23.7–111.8	0.003**	46.1	1.9 −90.2	0.041*
	Night shift numbers	132.7	−23.1–288.6	0.095	163.8	11.8–315.7	0.035*
Psychological (DASS-21) measures R²=0.016	Depression	16.6	−18.1–51.2	0.348	18.9	−14.5–52.4	0.266
	Anxiety	25.8	−9.2–60.8	0.148	10.8	−23.8–45.5	0.539
	Stress	−7.6	−39.5–24.4	0.643	−19.7	−52.4–12.9	0.236
Sleep quality (PSQI) measures. R²= 0.014	Global (PSQI)	77.2	24.9–129.5	0.004**	18.9	−43.8–81.9	0.553
12-Musculoskeletal symptom (NMQ) R² = 0.072	Number of pains sites last 12 months	256.2	182.7–331.2	0.001**	254.9	171.3–338.7	0.001**

BMI: Body Mass Index, PSQI: Pittsburgh Sleep Quality Index, NMQ: Nordic Musculoskeletal Questionnaire, DASS-21: Depression Anxiety Stress Scale, CIs: Confidence Intervals. R²: R Square, β: Regression coefficients.

* P < 0.05, **P < 0.01 (n = 597)

## Discussion

This study is the first to investigate physical activity levels and associated factors among Jordanian nurses. While previous research involving 3196 from the general Jordanian population reported that only 27% engaged in physical activity,^56^ our findings reveal a significantly higher proportion among nurses: 31% demonstrated moderate physical activity levels, and 40% exhibited high physical activity levels. This indicates that (71%) of nurses met or exceeded the recommended levels of physical activity, a notable contrast to the general population.

Interestingly, Jordanian nurses reported higher physical activity levels compared to their counterparts in countries like Canada, the United States, Spain, and Australia. For instance, only 41% to 50% of the U.S. nurses and 21% to 25% of nurses in Spain, Canada, and Australia met physical activity guidelines.^17,28,37,57,58^ This disparity may stem from environmental factors, the demanding nature of nursing work in Jordan, or the influence of workplace policies that promote physical activity, especially in governmental and military hospitals, where 85% of participants are employed. However, differences in physical activity measurement methods, such as whether occupational activity is included alongside leisure-time physical activity, may also explain these variations. Additionally, baseline physical activity levels can differ across countries due to cultural norms, infrastructure, and health policies, which should be considered when interpreting these findings.

Anthropometric and demographic models provided valuable insights into factors influencing physical activity levels. Notably, no significant associations were found between physical activity levels and factors such as age, sex, BMI, or the number of cigarettes smoked per day. The mean age of participants in our study was 32 years, which aligns with the younger age profile of the nursing workforce in Jordan.^59^ This is notably different from the mean age range of 42 to 48 years reported among nurses in Canada, the United States, Australia, and Spain.^17,28,37,57,58^ Previous studies have suggested that younger nurses are more likely to engage in work-related physical activity, potentially due to lower administrative responsibilities.^27^ McCarthy et al.^60^ and Almajwal et al.^26^ found that older nurses are less likely to meet recommended physical activity guidelines, possibly due to reduced physical job demands as they age. Therefore, the relationship between age and physical activity levels appears to be influenced by the nature of nursing duties and the demographic profile of the workforce, which vary across regions.

Concerning sex, some previous studies suggest that female nurses may engage less in physical activity due to additional domestic responsibilities and societal expectations,^22,61^ whereas other studies have found no significant sex differences in physical activity levels among nurses.^62^ The lack of association in our study may indicate that sex alone may not be a determining factor for physical activity among nurses in Jordan, and other contextual factors could play a more significant role. As for BMI, our finding of no association was somewhat unexpected, given the established link between higher BMI and lower physical activity levels in previous studies.^7,16,17,22,25^ For example, Al-Tannir et al.^63^ reported that nurses with a normal weight were independently associated with higher physical activity, while obesity predicted inactivity. The absence of a significant association in our study may be due to the physically demanding nature of nursing work in Jordan, which could lead to similar activity levels across BMI categories, or it may reflect cultural and occupational factors unique to this population. Additionally, self-reported measures of both BMI and physical activity may introduce bias, attenuating the observed association. Regarding smoking, previous studies found a negative association between smoking and physical activity, suggesting that smokers tend to be less physically active and engage in more sedentary behavior.^16,61^ One potential explanation for the difference in our findings could be the unique cultural and social factors influencing nurses in our study population. Additionally, the work environment and job demand specific to nurses could play a role in our results.^29,64^ For example, nurses may engage in more physical activity as a part of their job responsibilities, which could offset the sedentary tendencies often associated with smoking. Further research is needed to explore the relationship between tobacco and physical activity among nurses in diverse cultural contexts.

Our research revealed a negative association between the presence of at least one chronic disease and the level of physical activity among Jordanian nurses. The demanding nature of nursing work, including long hours, shift work, and high stress levels, can contribute to an increased risk of developing chronic health conditions in this population.^20,65^ Additionally, not participating in regular physical activity could increase the risk of chronic disease.^17,65^ Nurses are particularly susceptible to chronic conditions, such as obesity, musculoskeletal disorders, and cardiovascular disease.^17,66,67^ These findings underscore the need for comprehensive workplace wellness programs that address the specific needs of nurses and promote healthy lifestyle choices, including regular physical activity.^68^

We found a positive association between work hours per week and night shifts with physical activity levels. Different studies showed that night shift work is negatively associated with physical activity levels.^17,26,31^ In some work environments, night shifts might reduce physical activity due to disrupted circadian rhythms and increased fatigue, whereas in others, the demands of night shifts might still require considerable physical exertion.^64^ Our finding is consistent with the research by Peplonska et al., suggesting that nursing tasks require physical activity for an extended period.^29^ This activity could be due to the physical demands inherent in nursing tasks, which require nurses to be on their feet for prolonged periods, handle patients, and perform other physically intensive activities.^64,69^

The analysis showed no association between years of experience and physical activity levels. Different studies reported that more experienced nurses might engage less in physical activity due to increased administrative responsibilities and reduced physical demands of senior roles.^17,60,70^ The lack of association in our study may indicate that experience alone is not directly associated with physical activity levels. Instead, the nature of the job tasks and roles influences physical activity.

There were no associations between physical activity levels and measures of depression, anxiety, or stress among nurses. The scores of the Depression Anxiety Stress Scale indicated a moderate level of severity of depression, anxiety, and stress among participants, which is consistent with a previous study among U.S nurses that showed 35% have depression symptoms.^71^ Additionally, A notable prevalence of anxiety among nursing professionals has been observed, with studies indicating that rates range from 20% in Australian midwives^72^ to 46% in Iranian nurses.^73^ The lack of associations in our study is consistent with Freitas et al.^74^ suggested that the association between psychological well-being and physical activity among nurses may be influenced by a variety of mediating factors, such as job stress, shift work, and work environment. The lack of association in our study may reflect the overriding effect of occupational stressors or the possibility that job-related physical activity does not confer the same psychological benefits as leisure time activity.

Interestingly, our study found no association between Pittsburgh Sleep Quality Index (PSQI) scores and levels of physical activity, which contrasts with the research conducted by Lee et al.,^75^ which found a negative association between poor sleep quality and physical activity levels among Korean nurses. One explanation could be that participants who engage in higher levels of physical activity may experience better sleep quality. In essence, the positive effects of physical activity on sleep may mask any adverse effects of poor sleep quality on physical activity levels, leading to the lack of a relationship in our study. Alternatively, as seen in recent studies of Jordanian nurses, both sleep quality and physical activity levels were generally low, suggesting that other lifestyle or occupational factors may play a more dominant role in this population.^76,77^ Further research using objective measures and a longitudinal design is needed to clarify these relationships.

There was a positive association between the number of musculoskeletal pain sites in the past twelve months and physical activity levels. This indicates that nurses who reported more widespread musculoskeletal symptoms were also more active, which is somewhat counterintuitive given that pain is typically expected to discourage physical activity due to discomfort and limited activity.^22^ One possible explanation for the positive association is that nurses who engage in regular physical activity may experience better management of their musculoskeletal symptoms, potentially through strengthening muscles, improving flexibility, and enhancing overall physical fitness.^23,78^ It is also possible that nurses with higher physical activity levels are more susceptible to musculoskeletal symptoms due to the physically demanding nature of their work.^17,66^ Due to the cross-sectional design of the present study, we could not determine the directionality of this association. It would be beneficial to undertake longitudinal studies to establish whether higher physical activity levels will result in more or less musculoskeletal symptoms over time.

### Strengths and limitations

This study has several strengths. It used comprehensive regression models to analyze a wide range of factors that could influence physical activity levels among nurses, including anthropometric, demographic, work-related, psychological, sleep quality, and musculoskeletal pain variables. The large sample size and relatively high response rate improved the statistical power and reliability of the results. The use of standardized, validated measurement tools further supported the study's methodological rigor. Additionally, the research was conducted across multiple sites, which may enhance the representativeness of the sample.

However, several limitations should be acknowledged. The cross-sectional design restricts the ability to establish causal relationships between the associated factors and physical activity levels. Relying on self-reported measures introduces the possibility of recall bias and social desirability bias, which could affect response accuracy. Moreover, important variables such as dietary behaviors, environmental supports, or personal stressors were not measured and may have contributed to unexplained variance in physical activity outcomes. While the model included multiple domains, it explained only 12% of the variance in physical activity levels, suggesting that other factors such as the built environment, workplace culture, or access to physical activity resources need further investigation.

Moreover, the cultural specificity of the sample, comprising Jordanian hospital nurses, may limit the applicability of the results to other populations with differing healthcare systems, cultural norms, or occupational structures. Finally, the data were collected before the COVID-19 pandemic, a period that has since brought significant changes to the psychological well-being, workload demands, and sleep patterns of healthcare workers globally. As such, the findings may not fully reflect the post-pandemic realities of the nursing workforce, particularly in relation to mental health and physical activity behavior. These limitations emphasize the importance of future longitudinal and multicentre studies that include a wider variety of contextual factors and mirror changing healthcare settings, especially following COVID-19.

## Conclusions

This study highlights the physical activity levels among Jordanian nurses compared to both the general population and international nursing cohorts. Factors such as chronic disease, work characteristics, and musculoskeletal pain play a significant role in shaping physical activity levels. These insights can guide the development of tailored workplace interventions and policies to promote physical activity and overall well-being among nurses in Jordan. Addressing these factors can enhance nurses’ health and the quality of care they provide. Future research should explore these relationships longitudinally and in diverse cultural contexts to further inform evidence-based interventions.
